# LINKS BETWEEN LONELINESS AND HAPPINESS IN JAPAN’S AGING POPULATION

**DOI:** 10.1093/geroni/igz038.1130

**Published:** 2019-11-08

**Authors:** Takashi Yamashita, Anthony R Bardo, Jack Lam

**Affiliations:** 1 University of Maryland, Baltimore County, Baltimore, Maryland, United States; 2 University of Kentucky, Lexington, Kentucky, United States; 3 The University of Queensland, Indooroopilly, Queensland, Australia

## Abstract

Fertility rates in Japan have been historically low over several decades while life expectancy remains among the highest in the world. Consequently, traditional social networks consisting of immediate family and relatives have shrunk, and a growing number of older adults in contemporary Japanese society report feeling lonely. Thus, the well-being of Japans aging population is a major concern. While the negative effects of loneliness on perceived well-being (e.g., happiness) in later life have been well documented in western nations, relatively little is known from a Japanese context. Thus, we utilized a sample (n = 258) of urban community-dwelling Japanese adults age 65 years and older from the 2012 Survey of Mid-Life in Japan (MIDJA) to examine the association between happiness and loneliness. Consistent with findings from western nations, we identified strong links between happiness and loneliness in Japan. Results from ordinal logistic regression models showed that loneliness (OR = 0.80, p < 0.05) was negatively associated with happiness even after accounting for sociodemographic characteristics. Additionally, this study examined relevant demographic and cultural characteristics in order to contextualize the findings and identify possible explanations. For example, the cultural importance of family ties and gendered family roles was discussed in relation to the likely impact that increased levels of loneliness will have on the well-being of older Japanese adults. In sum, if the well-being of Japan’s rapidly aging population is to be maintained (or possibly even enhanced), then the growing societal issue of loneliness must be addressed.

